# Supplementary material for: Genome-wide identification and functional characterization of NPR1-like genes in Actinidia deliciosa
Source: PLoS One. 2025 Nov 13;20(11):e0334845. doi: 10.1371/journal.pone.0334845 (PMC12614555; doi:10.1371/journal.pone.0334845)
Supplement: S1 File — (DOCX) [file pone.0334845.s001.docx]

| Original Ids | Renamed Ids | Chr Number | Gene Position | | Number of amino acids | Molecular weight | pI Value | Instability index | Aliphatic index | GRAVY |
| --- | --- | --- | --- | --- | --- | --- | --- | --- | --- | --- |
|  |  |  | Start | End |  |  |  |  |  |  |
| Ademww1x09g132280.t1 | AdNPR1 | 9 | 1218394 | 1226457 | 593 | 65712.82 | 5.54 | 45.71 | 92.45 | -0.253 |
| Ademww1x01g003930.t1 | AdNPR2 | 1 | 4464271 | 4469900 | 583 | 64319.65 | 5.67 | 43.06 | 96.54 | -0.126 |
| Ademww1x14g219060.t1 | AdNPR3 | 14 | 18488691 | 18494386 | 589 | 65832.35 | 6.36 | 43.3 | 89.56 | -0.242 |
| Ademww1x23g350030.t1 | AdNPR4 | 23 | 6198391 | 6204970 | 586 | 65391.73 | 5.73 | 51.04 | 92.51 | -0.23 |
| Ademww1x19g294570.t1 | AdNPR5 | 19 | 20608575 | 20616792 | 588 | 65741.35 | 5.94 | 47.27 | 91.04 | -0.207 |

**Supplementary material Table S1**

**Accession Table 1**
