# Supplementary material for: Genome-wide identification and functional characterization of NPR1-like genes in Actinidia deliciosa
Source: PLoS One. 2025 Nov 13;20(11):e0334845. doi: 10.1371/journal.pone.0334845 (PMC12614555; doi:10.1371/journal.pone.0334845)
Supplement: Attachment — Submitted filename: Review Report.pdf [file pone.0334845.s002.pdf]

Review Report

Genome-Wide Identification and Functional Characterization of NPR1-Like Genes in Actinidia deliciosa

General Comments

This manuscript explores the genome-wide identification and characterization of *NPR1*-like genes in *Actinidia deliciosa*, aiming to understand their structural, evolutionary, and functional roles in plant defense. The topic is timely and relevant for crop improvement under biotic stress. The bioinformatics analyses are comprehensive, and the manuscript provides substantial insights. However, the manuscript requires several corrections and improvements to language, formatting, and clarity of scientific presentation.

Abstract and introduction

1. Ensure *NPR1* is italicized when referring to the gene and written in plain text when referring to the protein.

Results

2. Additionally, revise sentences like "Phylogenetic analysis revealed their evolutionary relationship with NPR1 homologs in model and crop species, suggesting functional divergence." to make them clearer and more concise. Avoid vague phrases such as "suggesting functional divergence" without context.

3. Clarify the gene naming criteria.

4. Why certain hits were excluded?

5. Ensure consistent naming of identified *AdNPR* genes.

6. Please provide rationale for rejecting genes lacking the NPR1-like C domain and explain the functional relevance of DUF3421 more clearly.

7. Interpret the phylogenetic tree more thoroughly by linking specific clades to potential functional conservation or divergence. Clarify what functional insights can be inferred from the grouping of *AdNPR* genes with *AtNPR* orthologs.

8. While motif patterns are shown, their functional implications are not discussed. Please identify which conserved motifs are essential for *NPR1* function and explain how exon/intron variability might contribute to gene regulation or alternative splicing.

9. Cis-element analysis is descriptive; please improve by discussing which transcription factors may bind these elements and how they might regulate gene expression under specific stresses or hormone treatments.

10. The evolutionary analysis would benefit from discussing how divergence times (e.g., 625 MYA) relate to known evolutionary events or species divergence in kiwifruit or related species. Please add context to why segmental duplications are important for gene family expansion.

11. Clarify why no synteny was observed with *A. chinensis*, despite its close relation to *A. deliciosa*. Is this due to annotation differences, sub-genome divergence, or data quality? A brief explanation will strengthen this analysis.

12. Expression profiling lacks statistical analysis. Please include fold-change values, p-values, or adjusted significance thresholds to validate gene expression changes under fungal and bacterial treatments.

13. The STRING PPI results suggest functional associations, but it is unclear which interactions are novel or confirm known pathways. Please highlight whether these predicted interactions validate known *NPR1*-mediated signaling or suggest new hypotheses.

Recommendations

14. **experimental validation** (qRT-PCR, mutant analysis) is required to well support your hypothesis.

15. Expression data is **purely descriptive**—no statistical testing or quantification of significance. Include statistical validation for expression data.

16. Clarify the objective behind each analysis (e.g., how does motif analysis inform gene function?).

17. Figures are not always well integrated with the text (some lack interpretation). Improve interpretation by linking findings to biological roles.

18. Discuss limitations and suggest future validation steps.
