# Supplementary material for: Genome-wide identification and functional characterization of NPR1-like genes in Actinidia deliciosa
Source: PLoS One. 2025 Nov 13;20(11):e0334845. doi: 10.1371/journal.pone.0334845 (PMC12614555; doi:10.1371/journal.pone.0334845)
Supplement: Attachment — Submitted filename: comments or openions.docx [file pone.0334845.s003.docx]

The manuscript Genome-Wide Identification and Functional Characterization of NPR1-Like Genes in Actinidia deliciosa has been written but needs some modifications as below

1. "five candidate genes (AdNPR1 – AdNPR5)" - It would be good to add here that they contain conserved BTB/POZ and ankyrin repeat domains, as this is a key finding (in abstract)

2. "Rasheed et al., 2025" - Please check all the publication years and ensure they are correct. There are quite a few 2025 citations.

3.  "Of these, gray mold caused by Botrytis cinerea is one of the most devastating diseases." - It might be helpful to briefly mention why gray mold is so devastating (e.g., yield loss, postharvest decay).

* Materials and Methods:

1. "Kiwifruit PanGenome Database (https://kiwifruitgenome.atcgn.com/) was used for BLAST-P program on 16th April 2025 at 18:23 Pakistan (UTC+5)" - While specific details are good, the time and date of the search might not be necessary. Consider removing it for brevity.

2. "Gene Pair file (prepared by using OpenAI tool)" - This is interesting, but it needs more explanation. How was OpenAI used to prepare this file? What kind of data was input, and what was the output?

* Results:

1. Figure 1 NCBI-CDD results: Showing the presence of..." - Improve figure captions to be more informative. Instead of "Showing the presence of...", describe what the reader should observe in the figure (e.g., "Domain structures of AdNPR genes, showing the presence of...").

2. Black dots are used to identify A. deliciosa." - This is too simplistic. The caption should explain what the phylogenetic tree shows in more detail (e.g., "Phylogenetic relationships among NPR1 homologs in A. deliciosa and other plant species..."). Also, it says "Black dots are used to identify A. deliciosa" but the black dots in Figure 2 are not labelled.

3. "Figure 3 shows the presence of AdNPR1 and AdNPR2 in cytoplasm in high concentrations..." - Be more specific about what "high" and "low" concentrations mean. Refer to the scale in the heatmap.

4.  "AdNPR2 has highest number of TATA-box in its promotor region as it serves as a recognition site..." - Rephrase for better flow: "AdNPR2 has the highest number of TATA-boxes in its promoter region, which serve as recognition sites...".

5. "Figure 7 Chromosomal mapping: The presence of AdNPRs genes on chromosome number1, 9, 14, 19, and 23." - Improve the caption. For example: "Chromosomal locations of AdNPR genes in A. deliciosa. Genes are located on chromosomes 1, 9, 14, 19, and 23." Also, in the caption, it says "chromosome number1" should it be "chromosome number 1"?

6. Discuss any limitations of the study and suggest potential directions for future research.

7. Domain Analysis: In Figure 1, you show the presence of different domains. Discuss the functional implications of the presence or absence of specific domains in the AdNPR genes.

8. Physicochemical Properties: While you present the physicochemical properties, discuss their biological relevance. For example, how might the GRAVY values or isoelectric points relate to protein function or localization

9. Phylogenetic Tree Interpretation: Expand on the evolutionary relationships revealed by the phylogenetic tree. Discuss the implications of the clustering patterns for the functional evolution of NPR1 genes in kiwifruit.
