# Supplementary material for: Genome-wide identification and functional characterization of NPR1-like genes in Actinidia deliciosa
Source: PLoS One. 2025 Nov 13;20(11):e0334845. doi: 10.1371/journal.pone.0334845 (PMC12614555; doi:10.1371/journal.pone.0334845)
Supplement: Attachment — Submitted filename: Revision.docx [file pone.0334845.s005.docx]

The article *“Genome-Wide Identification and Functional Characterization of NPR1-Like Genes in Actinidia deliciosa”* is well written. However, the authors should improve the overall English throughout the manuscript and carefully address the following suggestions:

1. There is no need to mention details such as “at 18:23 Pakistan (UTC+5)” or any similar date/region information anywhere in the manuscript. For example: “at 16:57 Pakistan (UTC+5).”
2. Please recheck the file extensions mentioned in line 214 (“.CTL, .Collinearity, and .GFF”) to confirm whether they should be in capital letters.
3. The text *“(Actinidia Lind.)”* in line 230 should be italicized.
4. In line 306, the caption reads: *“Color scale (0–18 WoLF-PSORT score) indicates predicted protein copies per cell; dark red = ≥15.”* It appears that a log scale has been used. Consider using a simple scale or revising the caption accordingly.
5. In line 325, *“such as drought.[2, 17]”* should be corrected to ensure the full stop comes after the reference.
6. In Figure 4, there is no need to include two images. A single heatmap would be sufficient from a publication perspective. Present it as a simple heatmap without the title “heatmap,” and add a boundary around it. Also, improve the figure quality, as the current version appears shrunk and unclear.
7. Replace the phrase *“Max. Numbers are of TATA-box”* with *“Maximum number of TATA-box.”*
